# Early Islamic glass (7^th^– 10^th^ centuries AD) in Unguja Ukuu, Zanzibar: A microcosm of a globalised industry in the early ‘Abbasid period

**DOI:** 10.1371/journal.pone.0284867

**Published:** 2023-06-07

**Authors:** Ieong Siu, Jianfeng Cui, Julian Henderson, Alison Crowther, Nicole Boivin, Elisavet Fergadiotou, Andrew Blair, Abdallah K. Ali, Simon Chenery

**Affiliations:** 1 Department of Anthropology, The Chinese University of Hong Kong, Shatin, Hong Kong; 2 School of Archaeology and Museology, Peking University, Beijing, China; 3 Department of Classics and Archaeology, University of Nottingham, University Park, Nottingham, United Kingdom; 4 School of Social Science, The University of Queensland, St Lucia, Brisbane, Australia; 5 Department of Archaeology, Max Planck Institute for the Science of Human History, Jena, Germany; 6 Perni, Chrysoupoli-Nestou, Kavala, Greece; 7 Department of Archaeology, Durham University, Durham, United Kingdom; 8 Ministry of Information, Tourism and Antiquities, Zanzibar Town, Zanzibar, Tanzania; 9 Inorganic Geochemistry, British Geological Survey, Keyworth, Nottingham, United Kingdom; New York State Museum, UNITED STATES

## Abstract

Eighty-two glass vessels, recovered from the excavations at the ancient Swahili settlement and port of Unguja Ukuu in Zanzibar, Eastern Africa, were analysed using laser ablation-inductively coupled plasma-mass spectrometry (LA-ICP-MS). The results show that all of the glass samples are soda-lime-silica glass. Fifteen glass vessels belong to the natron glass type and are characterised by low MgO and K_2_O (<1.50%), suggesting they were made from natron, a mineral flux that was widely used during the Roman period and Late Antiquity. Sixty-seven glass vessels belong to the plant ash glass type, characterised by high magnesia and potash levels (>1.50%), suggesting plant ash was the main alkali flux. Based on the major, minor and trace elements, three different compositional groups were identified for the natron glass and three were identified for the plant ash glass: (1) UU Natron Type 1, (2) UU Natron Type 2, (3) UU Natron Type 3, (4) UU Plant ash Type 1, (5) UU Plant ash Type 2 and (6) UU Plan ash Type 3. Comparison with contemporary Middle Eastern glass groups shows that UU Natron Types 1, 2 and 3 correspond to Egypt II high Na_2_O, Levantine I and Levantine II respectively, while UU Plant ash Type 1 matches closely with Samarra Group 2. UU Plant ash Types 2 and 3 have unique chemical fingerprints that do not match any of the contemporary plant ash glass groups, but their chemical compositions show some affinity with the old Sassanian plant ash glass, suggesting a possible Mesopotamian provenance. Combined with existing research on early Islamic glass, the authors reveal a complex trading network in the globalisation of Islamic glass, particularly involving glass corresponding to modern Iraq and Syria, in the 7^th^– 9^th^ centuries AD.

## Introduction

The investigation of early Islamic glass (7^th^– 10^th^ centuries AD) is a well-established field of material culture studies, particularly early Islamic glass in the Middle East. Scholars have conducted extensive research on Middle Eastern early Islamic glass using a cross-disciplinary approach (e.g. scientific, archaeological and typological approaches) and have generated important findings on issues concerning the provenance, production, distribution and consumption of early Islamic glass [1–14].

However, very little work has been done on early Islamic glass (especially glass vessels) in eastern Africa. The majority of research on glass in this region focuses either on glass beads or glass vessels from later periods [15–21] and much scientific research has focused on glass beads rather than vessels. Even though scholars have noted a typological relationship between glass vessels from eastern Africa (e.g. Kilwa, Shanga and Unguja Ukuu) and the Middle East, most studies of early Islamic glass vessels focus on art-historical and typological analyses [22–24]. For instance, Blair [24] has investigated the various functions of Islamic glass in Unguja Ukuu, Zanzibar and demonstrated the different roles Islamic glass played in a variety of functional and socio-economic contexts. However, he did not investigate the technology and provenance of Islamic glass, making it difficult to understand the possible sources of early Islamic glass and its movements between the Middle East and eastern Africa.

This state of affairs was partially remedied by three major studies using a cross-disciplinary approach to explore early Islamic glass beads and glass vessels from Unguja Ukuu in Zanzibar [25–27]. These researchers concluded that some of the natron and plant ash glass originated from the Syro-Palestinian region, Egypt and Iran/Iraq, and the glass beads and vessels could have been traded to eastern Africa from Iraq and Iran, via ports such as Siraf and Oman in the Persian Gulf.

Using electron microprobe analysis (EPMA), Fergadiotou [25] and Crowther *et al*. [26] have identified several glass compositional groups in the Unguja Ukuu assemblage and offered a general provenance of these glass groups, matching those from Samarra (Iraq), Nishapur (Iran) and al-Raqqa (Syria). Natron glass made in Egypt and the Levant can be separated into clear compositional groups according to the impurities introduced in the sands used to make it [9, 10]. It is possible to separate plant ash glass into broad compositional groups but using major and minor elements it can be difficult to define with confidence compositional subgroups which could reflect sub-regional production zones. For instance, although it is possible observe trends in the data which coincide with regions, it is nearly impossible to separate Levantine and Syrian glass with major and minor elements such as Al_2_O_3_, MgO, CaO, Na_2_O and K_2_O [7, 28].

With the use of sensitive techniques such as LA-ICP-MS and combined Sr-Nd isotopes [29], a clearer provenance definition can be identified. The research on Unguju Ukuu glass beads by Wood *et al*. [27] has shown some promising results on provenancing plant ash glass beads with trace elements and the merits of LA-ICP-MS in identifying raw materials used to produce glass and providing more refined provenance attributions for the Unguju Ukuu glass beads.

Accordingly, by using LA-ICP-MS to analyse glass vessels from 7^th^– 9^th^ centuries AD Unguja Ukuu in Zanzibar, we aimed to:

- identify the raw materials that were used to produce these glasses.
- identify the provenance(s) for early Islamic glass in Unguja Ukuu and to clarify whether they were locally made or imported using a combination of typological and scientific analyses.
- investigate the organisation and production of early Islamic glass from an eastern African perspective, with a particular focus on archaeological and scientific evidence.
- investigate the trading of early Islamic glass in the Islamic Middle East, the Indian Ocean and the Far East via the Silk Road.

## Materials and methods

### Archaeological site and glass samples

#### Unguja Ukuu and the glass samples

**Fig 1:**
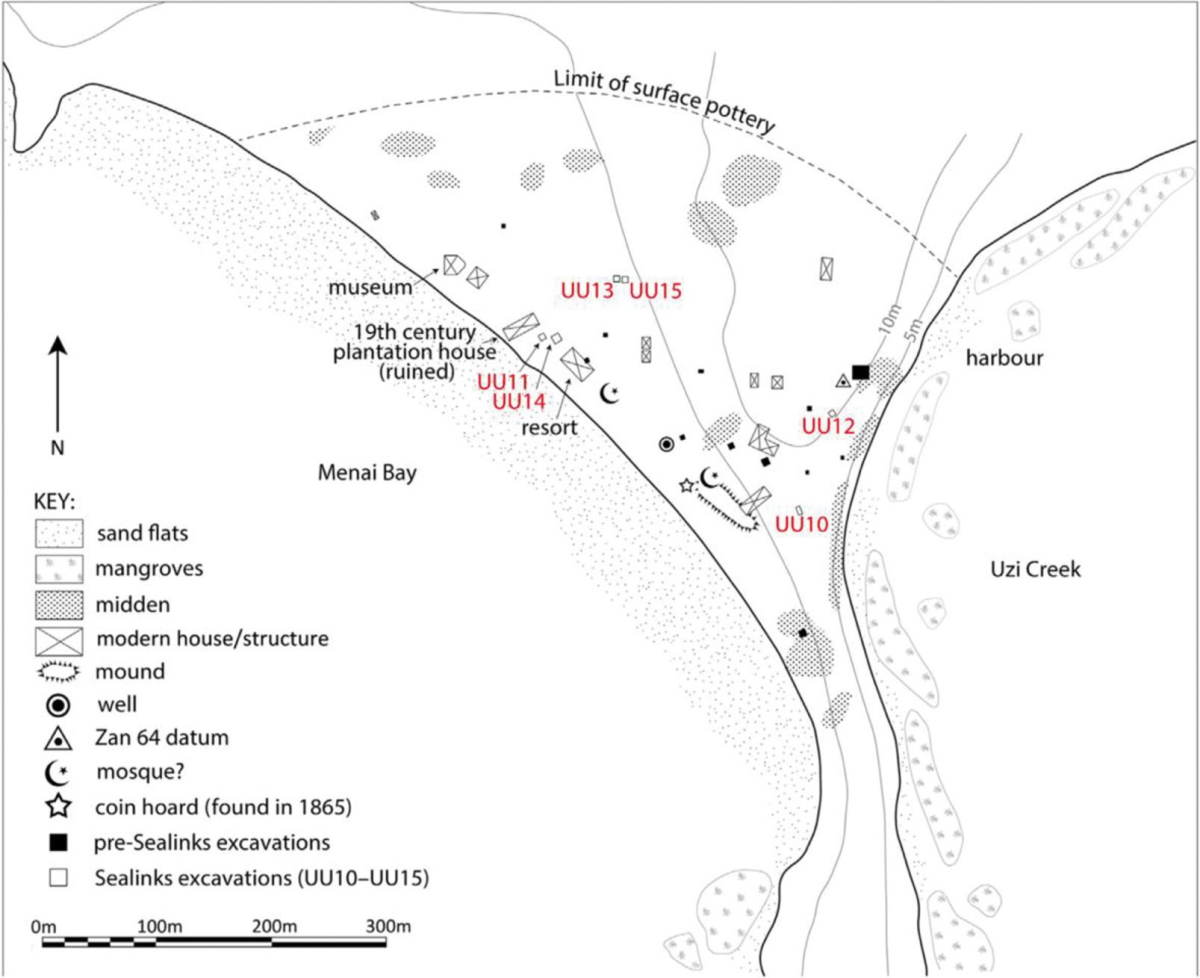
Unguja Ukuu site plan showing locations of excavated trenched. Most of the glass vessels came from Trenches UU10, UU11, UU13, UU14 and UU15.

The site of Unguja Ukuu has already been discussed extensively by Juma [30], Blair [24], Fitton and Wynne-Jones [31], Crowther *et al*. [26], and Fergadiotou [25]. Here we will offer a summary of the site and the archaeological contexts in which the majority of the glass remains were found. Unguja Ukuu is located in the southern part of the Unguja Island, the largest of the islands in the Zanzibar archipelago (Fig 1). It measures 87 km north to south and has an area of 1660 km^2^ [24, 30]. The settlement of Unguja Ukuu was founded in the 6^th^– 7^th^ centuries AD and subsequently developed into a bustling trading centre connected to long-distance maritime trade routes between the Middle East, the Persian Gulf and India in the 6^th^– 9^th^ centuries AD [24, 30, 31]. Islamic pottery (e.g. buff fabrics, eggshell ware), glass vessels, and South Asian glass beads were found and were most likely imported from regions like the Middle East, India and Sri Lanka [24, 27, 30]. By the 10^th^ century AD, however, Unguja Ukuu was in decline and the site was ultimately abandoned for reasons that remain unknown [24, 30].

In 2011–2012, the site of Unguja Ukuu was excavated as part of the Sealinks Project, which aimed to establish the early seafaring and long-distance connections between pre- and early historic communities occupying the Indian Ocean rim [24, 25, 32–35]. A total of 3625 glass fragments were excavated and recorded, of which eighty-two were selected for analysis and the authors have included all possible glass types and colours present in the assemblage. The glass samples are currently housed in the Department of Classics and Archaeology at the University of Nottingham, Nottingham, the United Kingdom, NG7 2RD. The specimen numbers for all samples are provided in the Finds Number column of Table 1. Permission is required for future access to the materials. All necessary permits were obtained for the described study, which complied with all relevant regulations. Permission to export the glass samples from Zanzibar to the UK for chemical analysis was granted by the Director of Museums and Antiquities, Zanzibar on 30/07/2011 (permit reference number: IMMK/MUK/16/VOL.I) and 13/09/2012 (permit reference number: IMMK/U/42/VOL.I/32).

**Table 1:** Description of the glass vessels from Unguja Ukuu.

Finds Number	Trench	Context	ID	Form	Type	Decoration	Date (century AD)	Colour
GL0038	UU10	002	Rim	Open	Stepped rim		Mid-7^th^– 8^th^	Olive green
GL0040	UU10	003	Rim	Open	Stepped rim		8^th^– 9^th^	Blue
GL0077	UU10	004	Base	Push-up	Edge of push-up		8^th^– 9^th^	Light green
GL0079	UU10	004	Rim	Open	Plain–rounded		8^th^– 9^th^	Ice blue
GL0090	UU10	004	Body	Undiagnostic	Undiagnostic	Scratch-engraved	8^th^– 9^th^	Blue
GL0119	UU10	004	Body	Undiagnostic	Undiagnostic		8^th^– 9^th^	Light green
GL0143	UU10	005	Rim	Semi-open	Vertical neck—wide		8^th^– 9^th^	Ice blue
GL0154	UU10	005	Rim	Open	Stepped rim		8^th^– 9^th^	Light green
GL0155	UU10	005	Rim	Open	Stepped rim		8^th^– 9^th^	Olive green
GL0190	UU10	006	Rim	Open	Inwards-folded rim		8^th^– 9^th^	Ice blue
GL0201	UU10	006	Misc	Application	Applied trail	Applied (trail)	8^th^– 9^th^	Colourless
GL0213	UU10	007	Misc	Application	Applied trail	Applied (trail)	8^th^– 9^th^	Olive green
GL0382	UU11	004	Rim	Semi-open	Vertical neck—wide		Mid-7^th^– 8^th^	Colourless
GL0459	UU11	005	Rim	Open	Plain–rounded		9^th^	Ice blue
GL0470	UU11	007	Body	Undiagnostic	Undiagnostic	Pinched	Mid-7^th^– 8^th^	Ice blue
GL0500	UU11	010	Base	Ring base	Applied ring-base		Mid-7^th^– 8^th^	Ice blue
GL0572	UU11	014	Rim	Closed	Neck C		Mid-7^th^– 8^th^	Ice blue
GL0635	UU11	014	Rim	Open	Stepped rim		Mid-7^th^– 8^th^	Olive green
GL0657	UU11	016	Base	Push-up	5		Mid-7^th^– 8^th^	Olive green
GL0659	UU11	016	Base	Push-up	3		Mid-7^th^– 8^th^	Olive green
GL0710	UU11	017	Body	Undiagnostic	Undiagnostic	Pinched	Mid-7^th^– 8^th^	Ice blue
GL0712	UU11	017	Body	Undiagnostic	Undiagnostic	Pinched	Mid-7^th^– 8^th^	Ice blue
GL0715	UU11	017	Body	Undiagnostic	Undiagnostic	Pinched	Mid-7^th^– 8^th^	Ice blue
GL0764	UU11	017	Body	Undiagnostic	Undiagnostic	Pinched	Mid-7^th^– 8^th^	Ice blue
GL0765	UU11	017	Body	Undiagnostic	Undiagnostic	Pinched	Mid-7^th^– 8^th^	Ice blue
GL0832	UU11	017	Body	Undiagnostic	Undiagnostic	Pinched	Mid-7^th^– 8^th^	Ice blue
GL0889	UU12	002	Rim	Open	Plain–rounded		9^th^	Ice blue
GL0904	UU13	006	Rim	Closed	Neck B		7^th^– 9^th^	Colourless
GL0911	UU13	006	Rim	Open	Plain–thick		7^th^– 9^th^	Colourless
GL0943	UU14	1412i	Rim	Open	Stepped rim		9^th^	Colourless
GL0949	UU14	1412i	Rim	Closed	Ribbed–narrow		9^th^	Ice blue
GL0976	UU14	1412h	Rim	Closed	Folded and flattened rim		9^th^	Ice blue
GL1014	UU14	1404e	Base	Push-up	6		9^th^	Ice blue
GL1029	UU14	1404e	Rim	Open	Stepped rim		9^th^	Ice blue
GL1030	UU14	1404e	Rim	Open	Plain–rounded		9^th^	Pale blue
GL1044	UU14	1404e	Base	Ring base	Folded ring-base		9^th^	Ice blue
GL1046	UU14	1404e	Base	Ring base	Folded ring-base		9^th^	Ice blue
GL1337	UU14	1404d	Rim	Semi-open	Vertical neck—wide		9^th^	Colourless
GL1339	UU14	1404d	Rim	Closed	Folded and flattened rim		9^th^	Turquoise
GL1343	UU14	1404d	Rim	Closed	Miniature jar		9^th^	Ice blue
GL1618	UU14	1406f	Rim	Open	Plain–rounded		9^th^	Ice blue
GL1620	UU14	1406f	Rim	Open	Plain–rounded		9^th^	Ice blue
GL1626	UU14	1406f	Base	Ring base	Folded ring-base		9^th^	Colourless
GL1633	UU14	1406f	Rim	Semi-open	Flaring—bevelled		9^th^	Ice blue
GL1637	UU14	1406f	Rim	Closed	Ribbed–narrow		9^th^	Ice blue
GL1638	UU14	1406f	Rim	Closed	Ribbed–narrow		9^th^	Olive green
GL1747	UU14	1403b	Rim	Open	Plain–rounded		9^th^	Ice blue
GL1853	UU14	1404e	Rim	Open	Plain–rounded		9^th^	Ice blue
GL1855	UU14	1404e	Body	Undiagnostic	Undiagnostic	Pinched	9^th^	Ice blue
GL1950	UU14	1406	Rim	Open	Triangular-beaked rim		9^th^	Colourless
GL2097	UU14	1423n	Rim	Closed	Folded and flattened rim		9^th^	Light green
GL2100	UU14	1423n	Rim	Closed	Neck A		9^th^	Light green
GL2147	UU14	1436	Rim	Open	Plate	Tooled	Mid-7^th^–late 8^th^	Turquoise
GL2244	UU14	1438	Rim	Open	Stepped rim	Scratch-engraved	Mid-7^th^–late 8^th^	Blue
GL2290	UU14	1431	Base	Push-up	5		Mid-7^th^–late 8^th^	Ice blue
GL2293	UU14	1431	Base	Push-up	Edge of push-up		Mid-7^th^ –8^th^	Olive green
GL2325	UU14	1418 W/S	Base	Open	Plate		9^th^	Blue
GL2450	UU14	1446	Rim	Open	Inwards-folded rim		Mid-7^th^–late 8^th^	Light green
GL2475	UU14	1443	Rim	Closed	Flaring—straight		Mid-7^th^–late 8^th^	Olive green
GL2478	UU14	1443	Rim	Open	Triangular-beaked rim		Mid-7^th^–late 8^th^	Light green
GL2522	UU14	1418L	Rim	Open	Plain–fine		9^th^	Ice blue
GL2580	UU14	1428 W/S	Body	Undiagnostic	Undiagnostic		Mid-7^th^–late 8^th^	Ice blue
GL2616	UU14	1408g	Rim	Open	Rolled-in rim		9^th^	Ice blue
GL2657	UU14	1418	Rim	Closed	Ribbed–narrow		9^th^	Ice blue
GL2677	UU14	1433	Base	Open	Plate		Mid-7^th^–late 8^th^	Blue
GL2689	UU14	1433	Rim	Semi-open	Flaring—bevelled		Mid-7^th^–late 8^th^	Light green
GL2715	UU14	1436 w/s	Base	Push-up	4		Mid-7^th^–late 8^th^	Olive green
GL2723	UU14	1421	Rim	Open	Plain–rounded		9^th^	Ice blue
GL2846	UU14	1420m	Body	Undiagnostic	Undiagnostic	Scratch-engraved	9^th^	Blue
GL2857	UU14	1440	Rim	Closed	Flaring—wide-mouthed		Mid-7^th^–late 8^th^	Ice blue
GL2933	UU14	1408 w/s	Body	Undiagnostic	Undiagnostic	Pinched	9^th^	Ice blue
GL2975	UU14	1414 or 1444	Misc	Application	Applied trail	Applied (trail)	Mid-7^th^–late 8^th^	Olive green
GL2978	UU15	1511j	Rim	Closed	Folded and flattened rim		7^th^– 9^th^	Light green
GL2980	UU15	1511j	Rim	Open	Plain–thick		7^th^– 9^th^	Olive green
GL2981	UU15	1511j	Rim	Open	Triangular-beaked rim		7^th^– 9^th^	Light green
GL3255	UU15	1556N	Rim	Closed	Flaring—straight		Mid-7^th^– 9^th^	Light green
GL3257	UU15	1556N	Misc	Application	Applied trail	Applied (trail)	Mid-7^th^– 9^th^	Olive green
GL3306	UU15	1510	Misc	Application	Applied trail	Applied (trail)	Late 10^th^– 11^th^	Olive green
GL3387	UU15	1511K	Rim	Closed	Flaring—wide-mouthed		7^th^– 9^th^	Olive green
GL3449	UU15	1551L	Rim	Open	Stepped rim		Mid-7^th^– 9^th^	Colourless
GL3450	UU15	1551L	Rim	Open	Stepped rim		Mid-7^th^– 9^th^	Colourless
GL3461	UU15	1551L	Misc	Application	Applied trail	Applied (trail)	Mid-7^th^– 9^th^	Turquoise

All glass vessels are dated to the mid-7^th^– 9^th^ centuries AD and come in different vessel forms and decorations.

**Fig 2:**
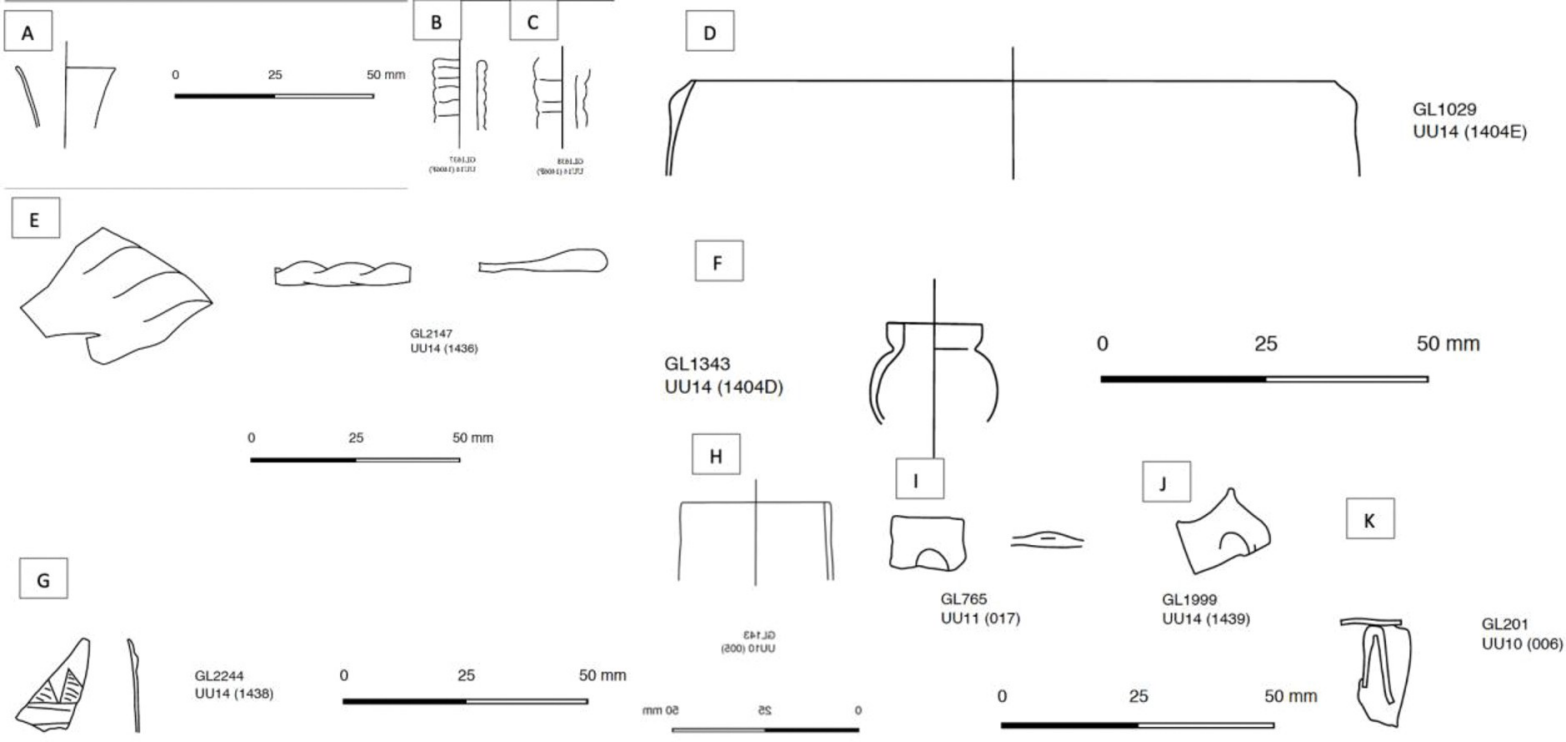
Examples of glass vessels from Unguja Ukuu. (A) Closed flaring–wide mouth GL2475; (B) Closed flaring–wide mouth GL3255; (C) Ribbed (narrow) GL1637; (D) Ribbed (narrow) GL1638; (E) Stepped rim GL1029; (F) Stepped rim GL0040; (G) Plate (tooled) GL2147); (H) Miniature jar GL1343; (I) Scratch-engraved GL0090; (J) Scratch-engraved GL2244; (K) Vertical neck–wide GL0143.

We have selected glass vessels that have diagnostic shapes in order to investigate the relationship between glass vessel types and compositions. They are broadly classified into four categories (Table 1; Fig 2), as devised by Blair [24]: (1) open rim types, which include plain rims (rounded, fine, thick), inward folded rims, stepped rims, triangular-beaked rims and splayed rims; (2) semi-closed and closed rim types, including folded and flattened rims, flasks with flaring necks, ribbed neck, jugs with vertical necks, flaring neck (bevelled rim, wide-mouthed, rolled-in, straight); (3) bases, which include different types of pushed-up bases, ring bases, and applied pad and ring bases; (4) decorated glass, including applied trail, scratch-engraved and pinched decorations. A variety of colours were also selected, including various shades of green and blue, turquoise and colourless. They are all dated to the 7^th^– 9^th^ centuries AD. This is based on the dating of the archaeological deposits and the majority of which are dated to the 7^th^– 9^th^/10^th^ centuries AD [24, 25]

The majority of the glass selected and analysed in this study came from trenches UU10, UU11, UU13, UU14, UU15 (Fig 1). Trenches UU 10 and UU13 were associated with waste dumping activity in the settlement. Trench UU10 was located to the south of the site on the edge of a small mound and consisted of two rubbish pits, Pits A and B, which indicate waste disposal activities in the area. Two radiocarbon dates for charred food residues in context 008 suggests Pit B is dated to 680–865 cal. AD and 670–840 cal AD. A total of 292 glass fragments were recovered in UU10 and the majority of them came from Pit B. The majority of the glass vessels from trench UU13 came from contexts 006 and 008 and are potentially dated to the 7^th^– 9^th^ centuries AD.

No structural remains were present in Trench UU11, but material remains provide ample evidence of occupation and trading activities. A total of 166 glass fragments were found in context 017, phase 2, which is dated to the mid-7^th^– 8^th^ century AD. Trench UU14, a possible site of both dumping and glass ‘transit zone’, was located 3 m to the northeast of UU11 and contained the largest number of glass fragments found in this project. A total of 2032 fragments were found and distributed across different phases of UU14, spanning from the earliest occupation layers (phase 2) to the modern dumping at surface level (phase 10). The largest quantity of early Islamic glass was found in phases 2 to 5. Finally, trench UU15 was located 4 m west of trench UU13. A series of postholes indicate the presence of timber structures in this part of the site, most likely dating to the 7^th^– 9^th^ centuries AD. The majority of the glass finds came from two main deposits in this trench, 1511 and 1551.

### Analytical methods

#### Laser ablation-inductively coupled plasma mass spectrometry

The glass samples were mounted in cold-setting epoxy resin, and then were grounded and polished using standard sample preparation procedures down to a 0.02 μm final polishing solution. The polished samples were analysed by laser ablation-inductively coupled plasma mass spectrometry (LA-ICP-MS) at the British Geological Survey in Keyworth, UK, to determine the major, minor and trace elements in the samples. The laser ablation unit was a NewWave (Electro Scientific Industries, Inc.) UP193nm ArFexcimer system operating at 193nm. The sample blocks were placed in a two-volume ablation cell with a 0.7 L min^−1^ He flow. In addition to the sample blocks, NIST glass standards SRM610 and 612, USGS GSD-1G and BCR-2G glass standards were placed in the chamber. For each analysis, the laser was fired at 10 Hz for 30 s using a beam diameter of 75 μm. Fluence and irradiance as measured by the internal monitor were typically 4.3 J/cm^2^ and 0.85 GW/cm^2^ respectively. Prior to introduction into the ICP-MS the He flow was mixed, via a Y-junction, with a 0.85 L min^−1^ Ar and 0.004 L min^−1^ N_2_ gas flows supplied by a Cetac Aridus desolvating nebuliser. The Aridus allowed introduction of ICP-MS tuning solutions and optimisation of the Aridus sweep gas (nominal 4 L min^−1^ Ar). During solid analysis by the laser, the Aridus only aspirated air. The ICP-MS used in this study was an Agilent 7500cs series instrument. The instrument was set for the 48 isotopes of interest, the dwell time for each isotope was 7 ms giving an integration time of 0.35s per time slice.

Data were collected in a continuous time resolved analysis (TRA) fashion. Prior to laser firing a period of at least 120 s of ‘gas blank’ was collected, then 3 ablations being made on each of the SRM610; SRM612; GSD-1G; BCR-2G; 3 ablations on up to 15 samples and finally a repeat of the ablations on the glass standards (for the results, see S1 Table). The SRM610 was used to calibrate the system for all elements except Mg, P, K and Fe which were calibrated using GSD-1G; whilst the SRM612 and BCR-2 were used as a quality control (QC) material. All calculations and data reduction were performed initially using Iolite 4 and from this data normalisation to 100% total oxides was made using the “Gratuze” method in Excel spreadsheets [36]. The precision of most elements for reference materials SRM612 and BCR-2G was typically good for both sessions (<5 RSD%), while the accuracies for most the elements ranged between -2 and +1%, which are considered within the range of quantitative results notable exceptions were P in SRM612 and Cu and Zn in BCR-2G, with poorer accuracy and precision (>10%).

### Results

The full LA-ICP-MS results are given in the S2 Table and the means and standard deviations for selected oxides and elements in the glass samples are presented in Table 2. The EPMA results are published elsewhere [25, 26]. We have used the EPMA results for major and minor element oxides (Na_2_O, MgO, Al_2_O_3_, SiO_2_, K_2_O, CaO and FeO) and the LA-ICP-MS results for other minor oxides and trace elements. All of the glasses are of the soda-lime-silica glass type. Using the oxides K_2_O and MgO, which usually derive from the alkali source, it is evident that fifteen glass vessels have low K_2_O (0.22% - 0.86%) and MgO (0.39% - 0.71%), while the rest of the glass vessels have high K_2_O (1.47% - 3.35%) and MgO (2.53% - 6.45%), indicating their sources of alkali are different. The former is derived from a mineral source (e.g. natron) and the latter from soda-rich plant ashes [4, 6]. We have identified five different glass groups based on the major, minor and trace elements.

**Table 2:** The means and standard deviations of Unguja Ukuu glass vessels.

Groups	UU Natron Type 1 (n = 3)	UU Natron Type 2 (n = 13)	UU Natron Type 3 (n = 3)	UU Plant ash Type 1 (n = 19)	UU Plant ash Type 2 (n = 34)	UU Plant ash Type 3 (n = 12)
Li (ppm)	3 ± 0.3	4 ± 0.8	7 ± 1.2	23 ± 6	16 ± 3	14 ± 3
B (ppm)	85 ± 61	57 ± 10	45 ± 2	94 ± 19	120 ± 24	137 ± 43
Na_2_O	15.86 ± 0.96	13.75 ± 0.77	12.86 ± 0.11	14.48 ± 0.93	15.20 ± 0.52	15.14 ± 0.53
MgO	0.42 ± 0.03	0.59 ± 0.07	0.57 ± 0.02	4.83 ± 0.77	3.66 ± 0.59	3.84 ± 0.44
Al_2_O_3_	2.07 ± 0.07	3.06 ± 0.28	3.33 ± 0.25	1.47 ± 0.22	2.55 ± 0.35	3.76 ± 0.23
SiO_2_	69.43 ± 0.54	70.80 ± 1.19	73.20 ± 0.70	68.50 ± 2.23	62.58 ± 1.75	59.29 ± 2.16
P_2_O_5_	0.05 ± 0.03	0.10 ± 0.04	0.04 ± 0.04	630 ± 169	1339 ± 1229	1355 ± 168
K_2_O	0.24 ± 0.03	0.61 ± 0.10	0.48 ± 0.04	2.82 ± 0.37	2.68 ± 0.35	2.19 ± 0.47
CaO	9.10 ± 0.57	8.85 ± 0.73	7.23 ± 0.34	4.99 ± 0.69	7.98 ± 1.00	10.06 ± 0.98
Ti (ppm)	1168 ± 78	481 ± 63	643 ± 117	557 ± 109	953 ± 134	1297 ± 143
V (ppm)	16 ± 0.1	10 ± 1	11 ± 2	13 ± 3	23 ± 5	25 ± 5
Cr (ppm)	19 ± 18	17 ± 2	16 ± 0.4	40 ± 9	82 ± 13	110 ± 19
Mn (ppm)	119 ± 4	247 ± 75	165 ± 19	3107 ± 3231	11523 ± 6092	8472 ± 9313
FeO	0.77 ± 0.04	0.59 ± 0.19	0.67 ± 0.19	0.65 ± 0.41	1.05 ± 0.24	1.42 ± 0.16
Co (ppm)	2 ± 2	2 ± 0.3	2 ± 0.4	72 ± 186	105 ± 256	7 ± 3
Ni (ppm)	6 ± 0.28	7 ± 1	5 ± 1	19 ± 5	32 ± 5	44 ± 8
Cu (ppm)	2 ± 0.11	156 ± 75	4 ± 0.4	129 ± 353	610 ± 2424	49 ± 43
Zn (ppm)	11 ± 27	23 ± 5	8 ± 1	174 ± 423	333 ± 772	56 ± 5
Rb (ppm)	3 ± 0.1	8 ± 1	8 ± 0.4	14 ± 2	13 ± 2	12 ± 3
Sr (ppm)	151 ± 7	441 ± 42	406 ± 4	386 ± 80	511 ± 90	529 ± 57
Y (ppm)	5 ± 0.4	7 ± 0.5	7 ± 0.3	4 ± 0.5	6 ± 0.5	7 ± 0.5
Zr (ppm)	113 ± 0.4	41 ± 4	50 ± 6	110 ± 24	103 ± 28	85 ± 20
Sn (ppm)	1 ± 0.1	52 ± 50	2 ± 0.1	6 ± 13	18 ± 31	7 ± 15
Cs (ppm)	0.04 ± 0.01	0.09 ± 0.02	0.05 ± 0.02	0.20 ± 0	0.21 ± 0	0.20 ± 0
Ba (ppm)	148 ± 2	261 ± 11	243 ± 2	146 ± 56	290 ± 150	240 ± 119
La (ppm)	6 ± 6	6 ± 0.4	7 ± 0.6	6 ± 0.9	8 ± 1	9 ± 1
Ce (ppm)	11 ± 0.3	12 ± 1	14 ± 1	12 ± 2	15 ± 1	17 ± 1
Nd (ppm)	5.5 ± 6.2	6.3 ± 0.3	7 ± 0.3	5 ± 0.7	6.7 ± 0.7	7.7 ± 0.5
Hf (ppm)	3 ± 0.1	1 ± 0.1	1 ± 0.2	3 ± 0.6	3 ± 0.6	2 ± 0.5
Pb (ppm)	2 ± 0.12	406 ± 378	8 ± 3	16 ± 27	263 ± 305	32 ± 83
Th (ppm)	1.2 ± 0	0.9 ± 0.1	1 ± 0.1	2 ± 0.2	2 ± 0.2	2 ± 0.2
U (ppm)	1.14 ± 0.8	0.81 ± 0.1	0.64 ± 0.03	0.65 ± 0.1	0.88 ± 0.1	0.95 ± 0.1

The results are presented according to the defined chemical compositional groups. Single samples are not included. Only selected oxides (wt %) and elements (in ppm) are presented here.

### Natron glass

**Fig 3:**
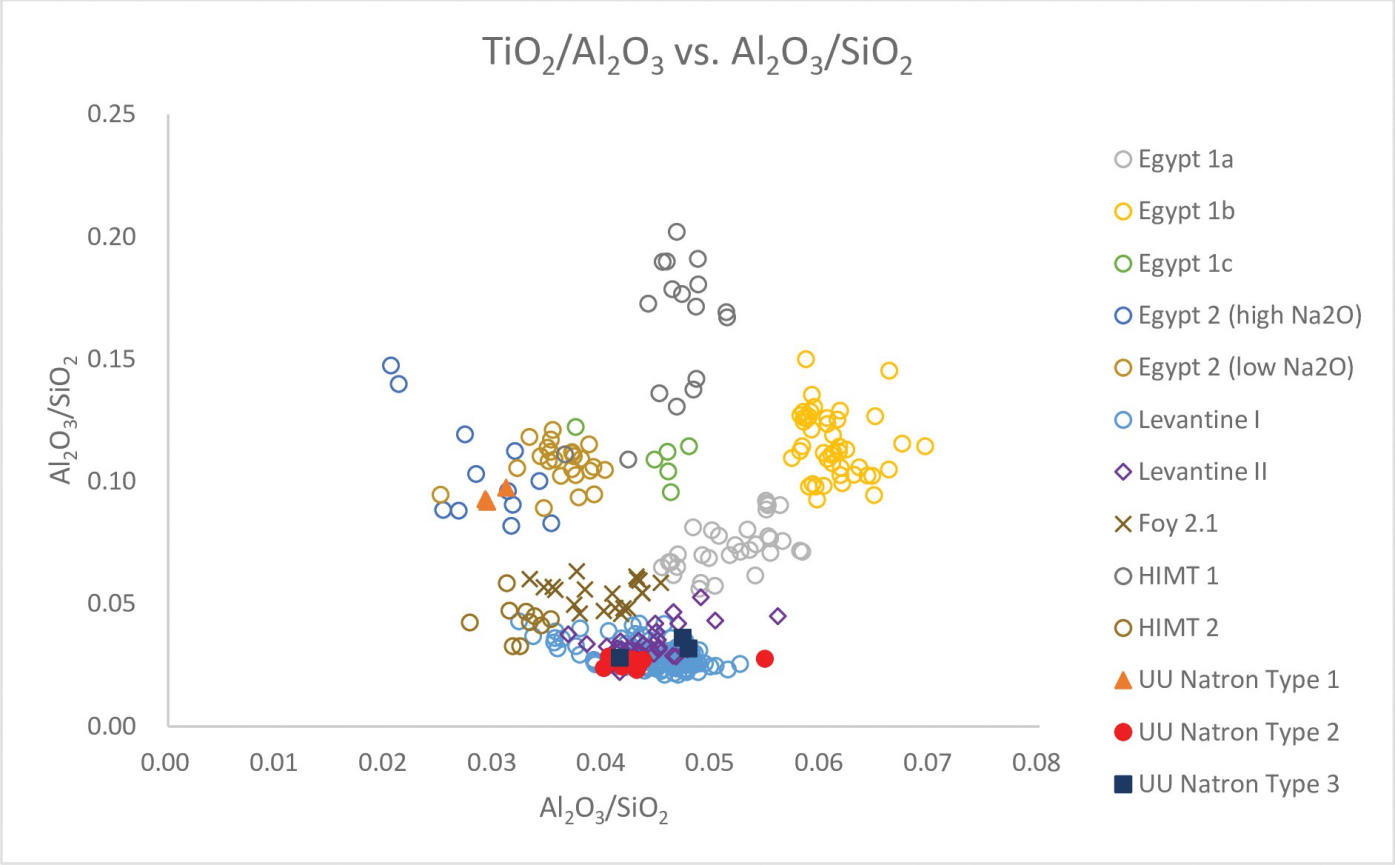
A biplot of TiO_2_/Al_2_O_3_ vs. Al_2_O_3_/SiO_2_ for Unguja Ukuu natron glass vessels with relevant 4^th^– 9^th^ centuries AD natron glass groups found in Egypt, Israel and Tunisia. The data is displayed according to compositional groups and sites. Data are from: Egypt 1a, 1b, 1c, Egypt 2 high Na_2_O and low Na_2_O [13]; Levantine I and Foy 2.1 glass from Khirbat al-Minya [38]; Levantine II glass from Ramla [10]; HIMT 1 and HIMT 2 glasses from Carthage [39].

**Fig 4:**
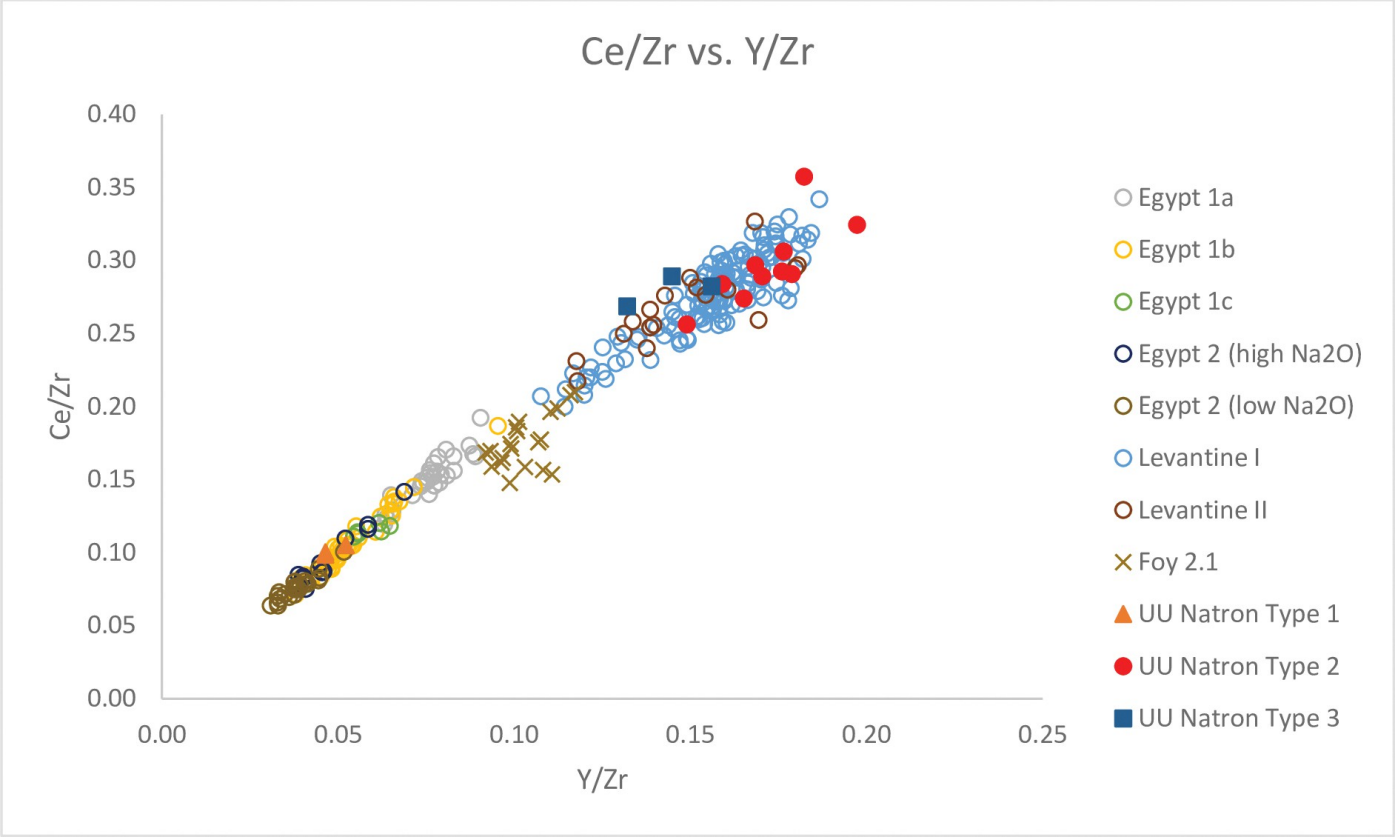
A biplot of Ce/Zr vs. Y/Zr for Unguja Ukuu natron glass vessels with relevant 4^th^– 9^th^ centuries AD natron glass groups found in Egypt and Syro-Palestine. The data is displayed according to compositional groups and sites. Data are from: Egypt 1a, 1b, 1c, Egypt 2 high Na_2_O and low Na_2_O [13]; Levantine I and Foy 2.1 glass from Khirbat al-Minya [38]; Levantine II glass from Ramla [10].

All natron glass are dated to the mid-7^th^– 8^th^ and 9^th^ centuries AD. They have K_2_O and MgO levels below 1.50%, which suggests natron was the primary alkali flux used to produce the glass. All glasses have relatively high levels of Al_2_O_3_ (2.02% - 3.81%), indicating the use of impure silica sources such as sands. They also have relatively high concentrations of CaO (7.15% - 10.15%), which derive from either shell fragments or limestone in sands [4, 37]. Figs 3 and 4 show that we can separate the Unguja Ukuu natron glass into two different compositional groups with elements associated with the silica source: major and minor oxides silica, alumina and titania, trace elements and Rare Earth Elements (REE) Zr, Ce and Y [12]. Lime and trace elements including Nd, Sr, Th and Ba are also useful to further illustrate the differences between each group [37].

### UU Natron Type 1

**Fig 5:**
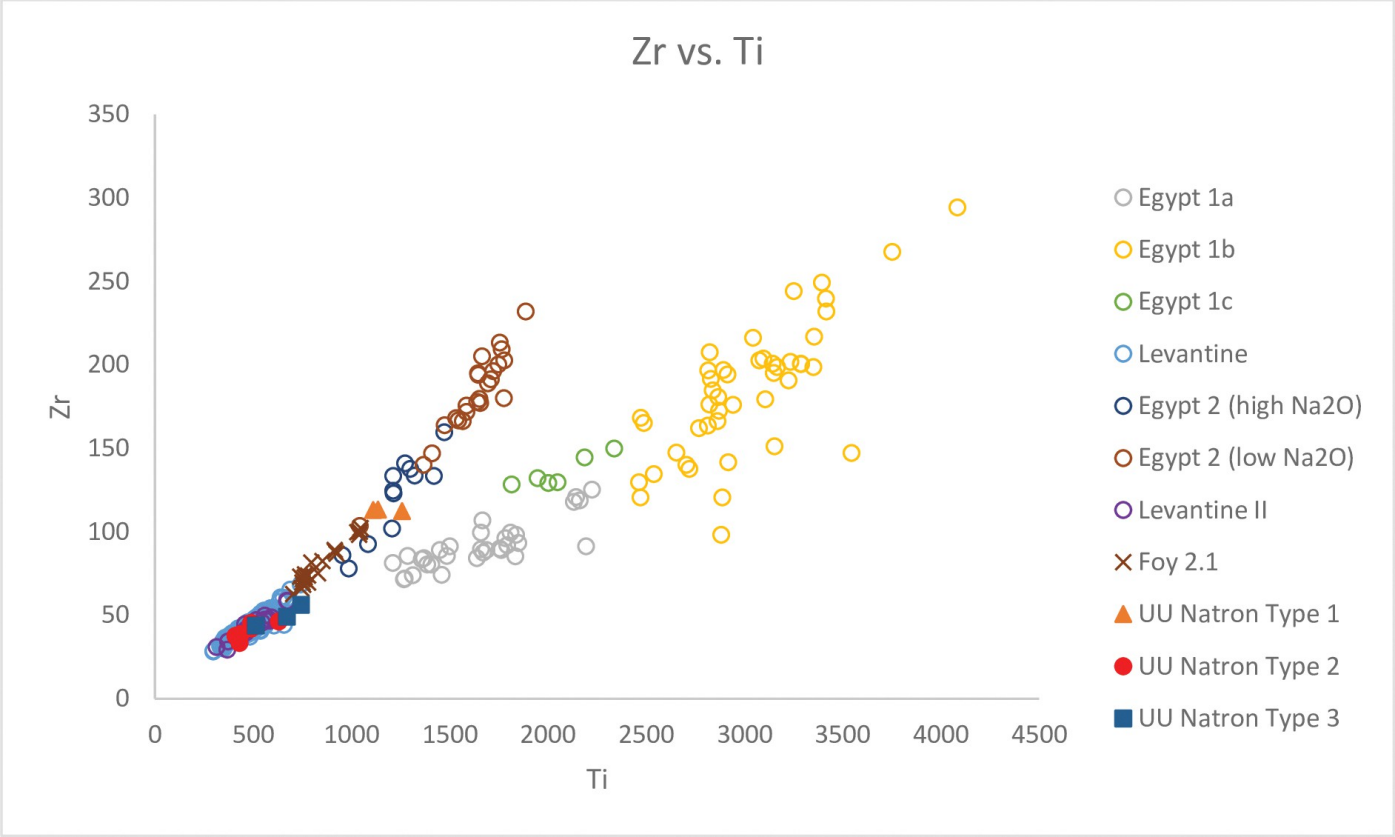
A biplot of Zr vs. Ti for Unguja Ukuu natron glass vessels with relevant 4^th^– 9^th^ centuries AD natron glass groups found in Egypt and Israel. The data is displayed according to compositional groups and sites. Data are from: Egypt 1a, 1b, 1c, Egypt 2 high Na_2_O and low Na_2_O [13]; Levantine I and Foy 2.1 glass from Khirbat al-Minya [38]; Levantine II glass from Ramla [10].

UU Natron Type 1 is characterised by high TiO_2_ to Al_2_O_3_ ratios and relatively low Al_2_O_3_ to SiO_2_ ratios (Fig 3). This group also contains high concentrations of CaO (8.44% - 9.43%), Ti (1111 ppm– 1257 ppm), Zr (112ppm– 113 ppm) and Hf (avg. 3 ppm) (Fig 5), which suggests quartz sands, which are rich in calcium carbonate and heavy accessory minerals like titanite, rutile and zircon, were likely used to make these glasses. The low concentrations of Sr (143 ppm– 155ppm) in the glass indicate the presence of limestone in the sands [37, 40]. UU Natron Type 1 also has a relatively low concentration of Ba (146 ppm– 149 ppm) and may partly have derived from limestones in sands [40]. The higher levels of REE such as Th (1.17 ppm– 1.20 ppm) and light REE such as La (5 ppm– 6 ppm), suggest they were introduced to the glass with heavy minerals like monazite [41].

### UU Natron Type 2

**Fig 6:**
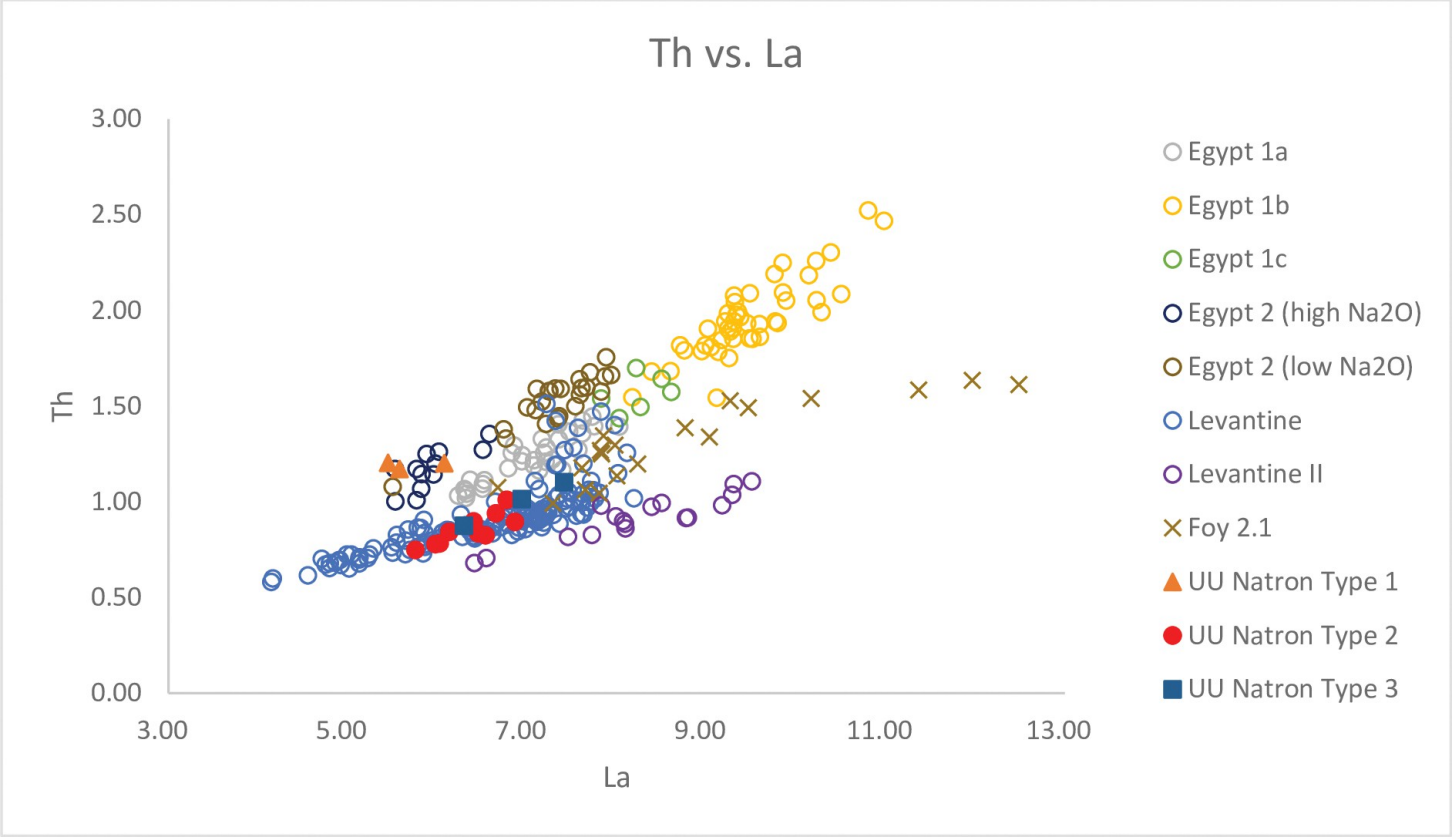
A biplot of Th vs. La for Unguja Ukuu natron glass vessels with relevant 4^th^– 9^th^ centuries AD natron glass groups found in Egypt and Israel. The data is displayed according to compositional groups and sites. Data are from: Egypt 1a, 1b, 1c, Egypt 2 high Na_2_O and low Na_2_O [13]; Levantine I and Foy 2.1 glass from Khirbat al-Minya [38]; Levantine II glass from Ramla [10].

This glass group has lower TiO_2_ to Al_2_O_3_ ratios and higher Al_2_O_3_ to SiO_2_ ratios compared to UU Natron Type 1 (Fig 3). There is also a strong positive correlation between Y to Zr and Ce to Zr ratios (Fig 4). The relatively low levels of Ti (430 ppm– 631 ppm), Zr (33 ppm– 46 ppm) and Hf (avg. 1 ppm) suggest sand sources with lower heavy accessory minerals were used to produce these glasses. But the higher concentrations of Al_2_O_3_ (2.86% - 3.81%) suggest that these were mature high silica sands with high feldspar contents [10]. The levels of CaO (8.13% - 10.15%) and Sr (388 ppm– 522 ppm) are notably high, and a strong positive correlation is observed between the two, indicating that lime in the glass was most likely derived from shell fragments in coastal sand [37]. Th and La are often associated with heavy minerals such as monazite and allanite. The high and positively correlated Th and La (Fig 6) suggests they were likely introduced with heavy minerals such as monazite and allanite [40].

### UU Natron Type 3

This glass group is characterised by relatively low TiO_2_ to Al_2_O_3_ ratios and high Al_2_O_3_ to SiO_2_ ratios (Fig 3). What distinguishes UU Natron Type 3 from UU Natron Type 2 are the lower ratios of Y/Zr (Fig 4) and Na_2_O (12.74% - 12.89%) levels and the higher concentrations of SiO_2_ (72.40% - 73.66%) and La (avg. 7 ppm), suggesting that different sand sources were used to produce UU Natron Type 3 glasses.

### Plant ash glass

All plant ash glass in the Unguja Ukuu glass assemblage is characterised by high MgO and K_2_O exceeding 1.5 weight %, showing that soda-rich halophytic plant ashes were used. The levels of Al_2_O_3_ and Ti vary between 1.04% and 4.34%, and between 419 ppm and 1528 ppm respectively. The content of CaO also varies significantly, ranging from 4.28% and 10.95%. We found that major and minor oxides such as MgO, CaO, Al_2_O_3_ and Ti, and trace elements associated with the silica source, Cr, La, Zr and Ce, and those associated with the alkali source, Cs and Li [8], are the most effective discriminators in separating plant ash glass into different groups. Three main groups were found: (1) UU Plant ash Type 1, (2) UU Plant ash Type 2 and (3) UU Plant ash Type 3.

### UU Plant ash glass Type 1

**Fig 7:**
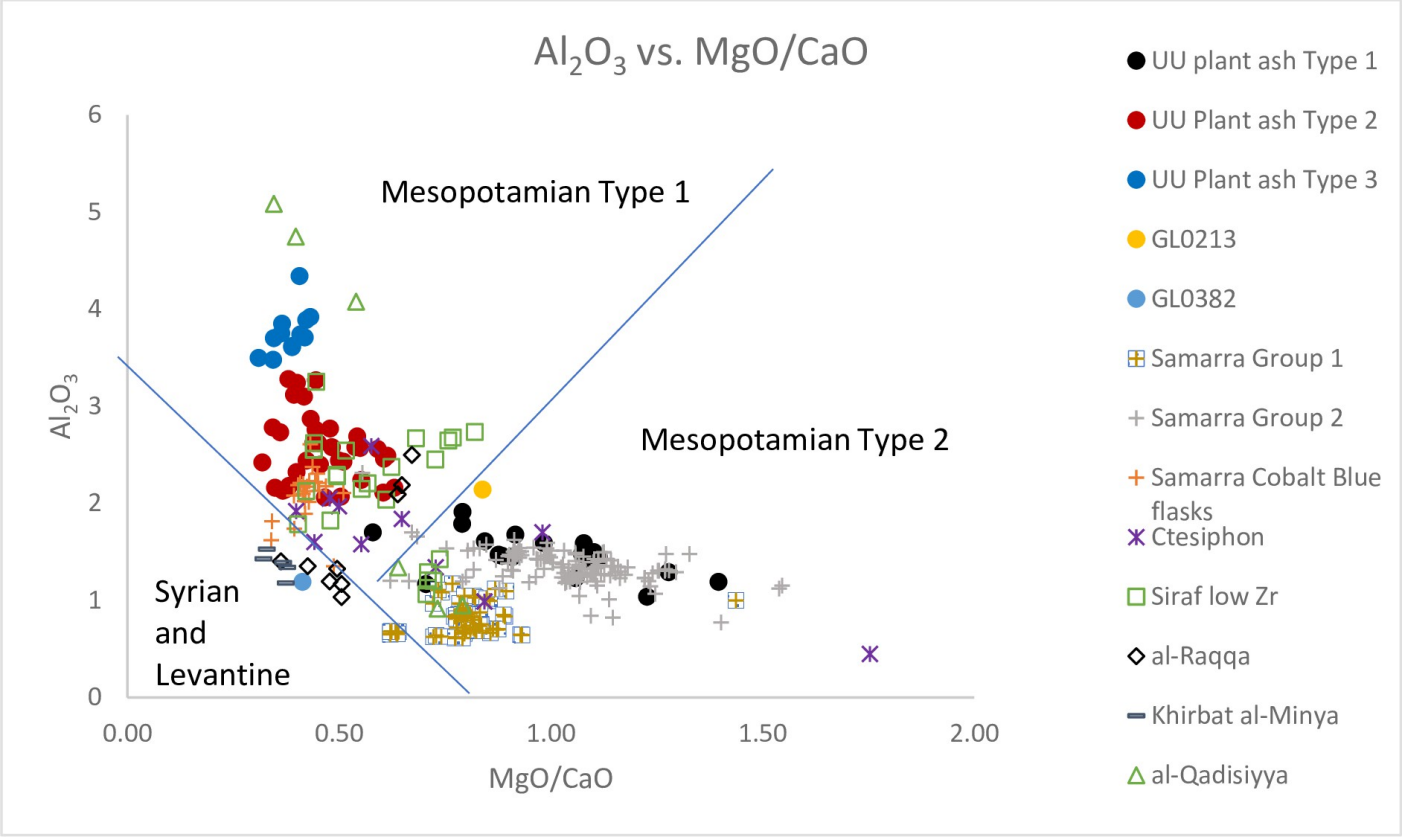
A biplot of Al_2_O_3_ vs. MgO/CaO for Unguja Ukuu plant ash glass vessels with relevant 4^th^– 9^th^ centuries AD plant ash glass groups found in Israel, Syria, Iraq and Iran. The data is displayed according to compositional groups and sites. Data are from: Samarra Groups 1 and 2 and Cobalt Blue flasks [12]; Ctesiphon, al-Raqqa, Nishapur and Khirbat al-Minya [7]; Siraf Low Zr glass [42]; al-Qadisiyya [43].

**Fig 8:**
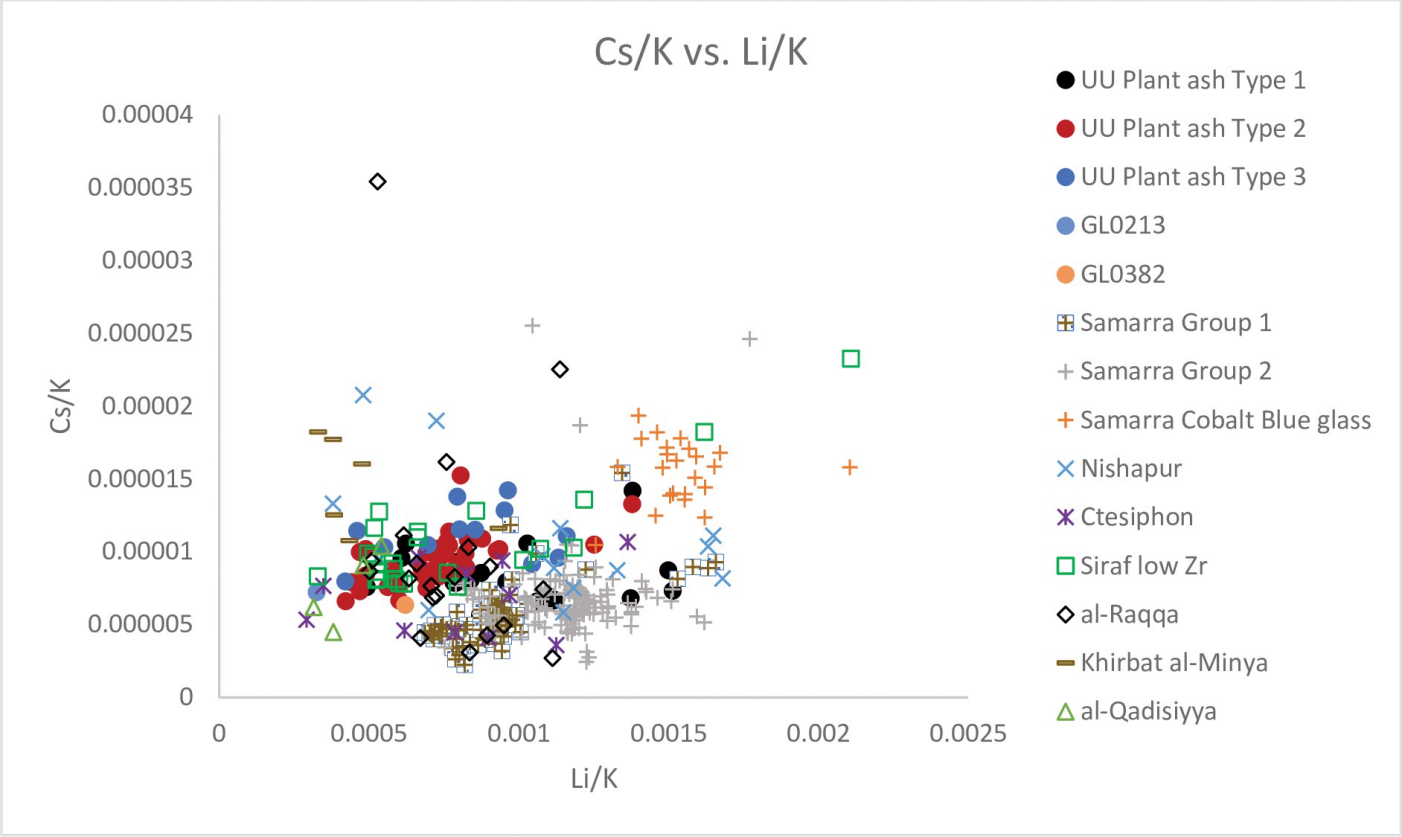
A biplot of Cs/K vs. Li/K for Unguja Ukuu plant ash glass vessels with relevant 4^th^– 9^th^ centuries AD plant ash glass groups found in Israel, Syria, Iraq and Iran. The data is displayed according to compositional groups and sites. Data are from: Samarra Groups 1 and 2 and Cobalt Blue flasks [12]; Ctesiphon, al-Raqqa, Nishapur and Khirbat al-Minya [7]; Siraf Low Zr glass [42]; al-Qadisiyya [43].

UU Plant ash Type 1 is characterised by low concentrations of Al_2_O_3_ (1.04% - 1.91%) and Ti (351 ppm– 704 ppm) (Fig 7; S2 Table) and relatively high concentrations of SiO_2_ (62.04% - 70.63%). The levels of trace elements associated with the silica source, such as Cr (29 ppm– 56 ppm) and La (3 ppm– 6 ppm) are also fairly low. This suggests a relatively pure source of silica such as crushed quartz pebbles were used [12]. The majority of UU Plant ash Type 1 glass has distinctively high MgO to CaO ratios (0.58–1.40) and Li to K ratios (Figs 7 and 8).

### UU Plant ash glass Types 2 and 3

These two groups are compositionally different from UU Plant ash Type 1. They contain higher concentrations of Al_2_O_3_ (2.07% - 4.35%) and Ti (621 ppm– 1528 ppm), and REE and trace elements such as Cr (60 ppm– 137 ppm) and La (5 ppm– 10 ppm) compared to UU Plant ash Type 1. This indicates the use of a relatively impure source of silica, perhaps quartz sands contain heavy minerals [4, 12]. The lower MgO to CaO ratios and Li to K ratios also suggest different sources and/or species of plants were used to produce glass in UU Plant ash Types 2 and 3 (Figs 7 and 8).

What distinguishes UU Plant ash Types 2 from UU Plant ash Type 3 is the higher concentrations of Al_2_O_3_ (exceeding 3.50%), Ti (exceeding 1200 ppm), and silica-related REE and trace element Ce (>15 ppm) and Cr (avg. 110 ppm) in UU Plant ash glass Type 3, showing that a different silica source defined by higher contents of heavy accessory minerals was used. It is also noted that glass from UU Plant ash Types 2 has higher concentrations of Mn (avg. 11523 ppm compared to avg. 8472 ppm in UU Plant ash Type 3), which was most likely added to the glass deliberately as a colouring or de-colouring agent to Islamic glass [6, 12].

### GL0213 and GL0382

**Fig 9:**
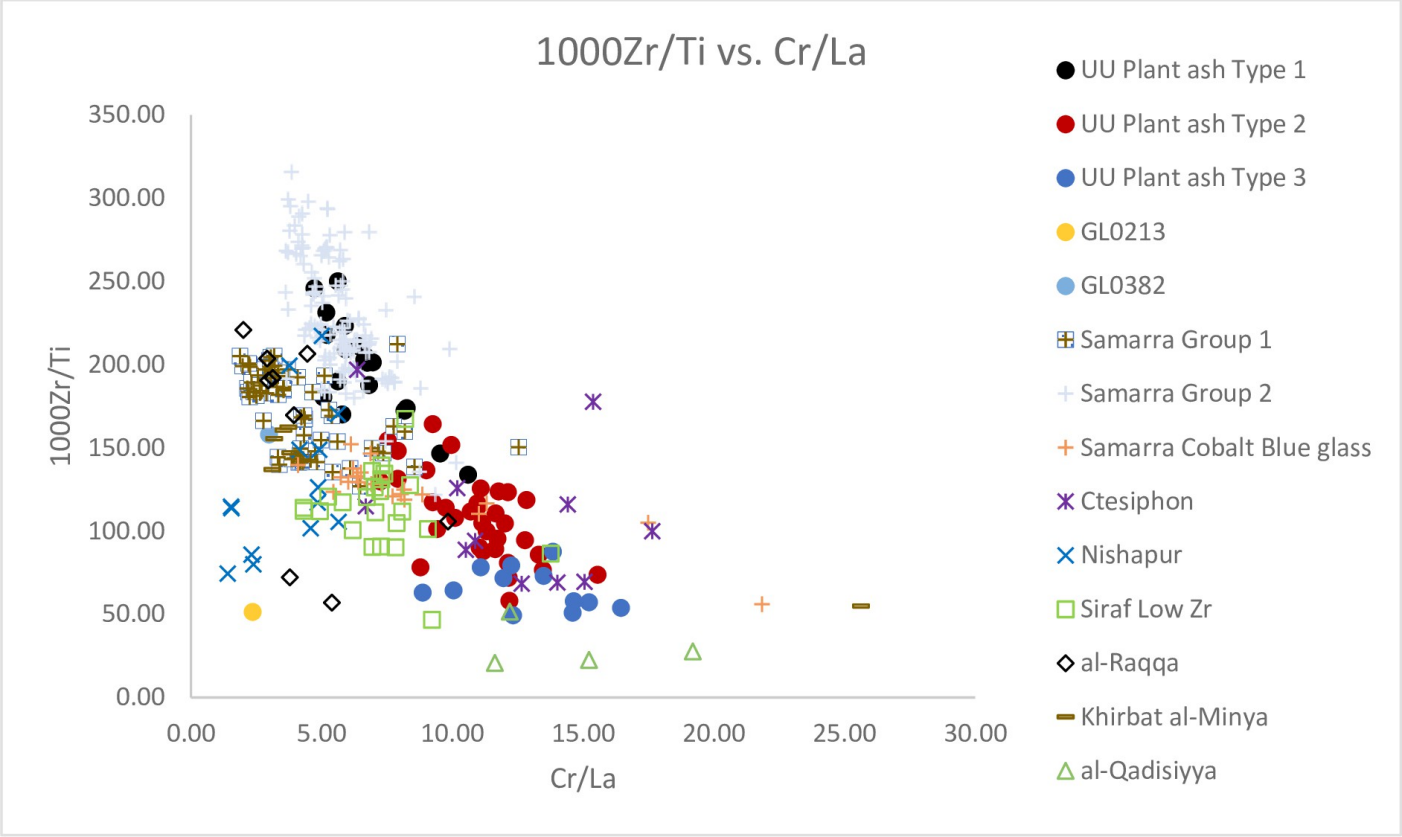
Biplot of 1000Zr/Ti vs. Cr/La for Unguja Ukuu plant ash glass vessels with relevant 4^th^– 9^th^ centuries AD plant ash glass groups found in Israel, Syria, Iraq and Iran. **A** The data is displayed according to compositional groups and sites. Data are from: Samarra Groups 1 and 2 and Cobalt Blue flasks [12]; Ctesiphon, al-Raqqa, Nishapur and Khirbat al-Minya [7]; Siraf Low Zr glass [42]; al-Qadisiyya [43].

An olive-green applied trail vessel (GL0213) and an ice blue pinched vessel (GL0382) are compositionally different from other plant ash glass groups. GL0213 appears to be different with higher levels of Al_2_O_3_ (2.14%), P (11565 ppm), Ti (1463 ppm) and B 174 ppm) and lower levels of Cr (18 ppm) and Li (6 ppm) than the average levels found in UU Plant ash Type 1. On the other hand, GL0382 has low levels of Al_2_O_3_ (1.19%), Cr to La ratios and 1000Zr/Ti ratios (Fig 9). It is likely that they were manufactured with sand sources different from other plant ash glass groups studied here.

### Glass colouring

All but one natron glass are an ice blue colour. Glass GL2975 has an olive-green colour. All glasses have high concentrations of FeO (0.42%– 0.94%), which was most likely introduced as impurities in the silica source [44, 45]. The pale blue colour is produced by the presence of iron in the glass often as ferrous ion (Fe^2+^), while the olive colour is most likely produced by iron presented as ferric ion (Fe^3+^) [44, 46].

Plant ash glasses were produced in a wider range of colours than the natron glass. They are coloured in ice blue, blue, olive and light green and turquoise, and some of which are colourless. The ice blue glass vessels contain high levels of FeO (0.33%– 1.57%); the light blue colour is due to the presence of iron in in its reduced form (Fe^2+^). The high iron oxide contents (1.07% - 1.91%) in blue coloured glass (GL40, GL90, GL2325 and GL2846) suggests that iron was deliberately added to the glass to produce the blue colour. On the other hand, the blue colour in a scratch-decorated vessel with a stepped rim (GL2244) was likely produced by copper (14215 ppm) in cupric ion (Cu^2+^) [37, 44].

Glass vessels coloured in olive and light green, and turquoise have high concentrations of FeO (0.40% - 1.75%), indicating that iron was deliberately added to the glass to produce different shades of green. The green colour was likely to have been produced by the presence of mixed ferric (Fe^3+^) and ferrous (Fe^2+^) ions [44, 46].

All of the colourless glass has significant amounts of Mn (4647 ppm– 24455 ppm), which suggests manganese was deliberately added to the glass. Manganese is a well-known decolouriser in the early Islamic Middle East. Its use was mentioned by the Islamic chemist Jabir Ibn Haiyan who wrote about the use of manganese dioxide in glassmaking [6].

## Discussion

### Natron glass

Natron glass was produced between around 3800 BC and AD 800 in the Syro-Palestinian region and Egypt. Recent research has identified seven main compositional groups and subgroups of natron glass based on major and minor oxides: (1) Levantine I; (2) Levantine II; (3) Egypt 1a, 1b and 1c; (4) Egypt 2 high Na_2_O and low Na_2_O; (5) HIMT; (6) Foy 2.1; and (7) Foy 3.2 [3, 11, 13, 39, 47–49], with some refinements for natron glasses used in the iron age [40].

Using a simple biplot of Ce/Zr versus Y/Zr, we can separate natron glass into two broad regional groups, Levantine and Egyptian groups. UU Natron Type 1, consists of a ribbed bottle neck (narrow) (GL1637) and a push-up base (GL2290), corresponding to glass groups made in Egypt, which is characterised by low Y to Zr and Ce to Zr ratios (Fig 4) [50]. TiO_2_/Al_2_O_3_ and Al_2_O_3_/SiO_2_ ratios are better discriminants in distinguishing between different groups of Egyptian glass. Fig 3 shows that UU Natron Type 1 is consistent with Egypt 2, which have low Al_2_O_3_ to SiO_2_ ratios and high TiO_2_ to Al_2_O_3_ ratios. Overall, Egypt 2 glasses have lower minor and trace concentrations of Ti, Sr and La but higher concentrations of CaO than Egypt 1 glasses (Figs 5 and 6). They also contain high concentrations of trace elements and REE such as Zr, Th and Hf.

Schibille et al. [13] have identified two subgroups of Egypt 2 based on the differences in the concentrations of Na_2_O. UU Natron Type 1 is identified with the subgroup ‘Egypt 2 high Na_2_O’ with an average Na_2_O level of 15.86%, which compares with an average of 13.50% Na_2_O in ‘Egypt 2 low Na_2_O’ glass [13]. It is also notable that Egypt 2 high Na_2_O glasses generally have lower levels of trace elements and REEs such as Zr, Th, La and Hf (Figs 4–6), reflecting the use of a different silica source characterised by low trace elements and REEs probably involving a different glassmaking technology. UU Natron Type 1 does not appear to be associated with any of the HIMT glass groups, including recycled glasses found in southern and northern Europe (e.g. Italy) [51]. HIMT and its recycled types were very popular in the late Roman period and late antiquity and were most likely made in Egypt. HIMT is characterised by higher TiO_2_/Al_2_O_3_ and Al_2_O_3_/SiO_2_ ratios (Fig 3). A recent study of early Islamic Egyptian glass weights has dated the production of Egypt 2 to between AD 760/780 and 870 in Egypt [13]. Many Egypt 2 glass vessels found in Egypt, such as those from Tebtynis and Fustat, are also dated to the late 8^th^– 9^th^ centuries AD [13, 52].

The locations of Egypt 2 glassmaking workshops are currently unknown. The low Sr relative to CaO contents in the glass suggest the sources of lime came from inland sand containing limestone [37]. The strontium isotope study of Egypt 2 glass from the secondary glassworkshop at Ashmunein in Egypt suggests the lime-rich particles in the sand used to make the Ashmunein glass derived from Oligocene or late Eocene limestone, which occurred in northern Egypt, such as around the latitude of modern Cairo [37]. It is possible that the production location of Egypt 2 was in northern Egypt around the Cairo region. The relatively high concentration of Na_2_O could suggest that the glass workshop was not too far away from sources of natron deposits such as the lakes of Wadi Natrun, which is located about 100 km north-west of Cairo [53], though this depends on the technological procedures used.

UU Natron Types 2 and 3 form the bulk of the natron glass in this study and are consistent with glasses from the Levantine region. It is a fairly homogenous group with little compositional variation. Compared with the Egyptian glass groups, Levantine glasses have higher Ce to Zr and Y to Zr ratios (Fig 4). They also have rather high concentrations of Al_2_O_3_ and low levels of the minor oxides MnO and FeO and trace elements Zr, Ti and Th (Figs 5 and 6), suggesting the use of a rather mature sand source characterised by a low content of heavy minerals and a high content of feldspar [10]. Compared to Egypt 1 and 2, the UU Natron Types 2 and 3 glasses have a higher concentration of Sr relative to CaO. The high concentrations of Sr in the glass suggest lime was derived from aragonitic mollusc shells in coastal sand [37], probably at or near the mouth of the Belus River, which contained a 15% calcium carbonate content and can provide a level of about 8% CaO within typical natron glass [54, 55].

It is possible to distinguish between different groups of Levantine glass based on the concentrations of CaO, Na_2_O, Al_2_O_3_, SiO_2_ and La. Levantine I glass is characterised by higher CaO and Al_2_O_3_ contents than Levantine II glasses. Levantine II glasses contain lower Na_2_O and higher levels of SiO_2_ and La (Fig 6) than Levantine I glasses [3, 56]. Based on these criteria, it is noted that UU Natron Type 2 glasses fall into the Levantine I group, while UU Natron Type 3 glasses are consistent with the Levantine II group.

Excavations of primary glass production centres in Apollonia and Dor have yielded huge amounts of Levantine I raw glasses, which confirm its origin was in the Syro-Palestinian region [39, 48]. By the late 7^th^– 8^th^ centuries AD, Levantine II glasses appeared and replaced Levantine I glasses in the region, with a production centre located at Bet Eli’ezer [3]. Given that most of the Levantine I production centres ceased to exist by the 7^th^ century AD and the majority of the UU Natron Type 2 glasses have slightly elevated levels of Pb (avg. 328 ppm) and Cu (avg. 121 ppm), these vessels were probably made using recycled Levantine I glass that had been in existence for around 200 years, though they do not have typical compositional characteristics indicating that they had been mixed/recycled. On the other hand, the low levels of Pb (avg. 8 ppm) and Cu (avg. 4 ppm) in UU Natron Type 3 glass indicate that fresh Levantine II glass continued to be produced and exported in small quantities to regions as far as East Africa between 7^th^ and 9^th^ centuries AD.

### Plant ash glass

Using a biplot of Al_2_O_3_ vs. CaO/MgO ratios (Fig 7), we can separate early Islamic plant ash glasses into three different broad regional groups. Syro-Palestinian glasses (e.g. al-Raqqa and Khirbat al-Minya) contain low MgO/CaO ratios and low levels of Al_2_O_3_ and ‘Mesopotamian’ glasses (i.e. those found in Iraq and Iran) generally contain higher MgO/CaO ratios with regional groups definable based on Al_2_O_3_ levels: ‘Mesopotamian’ Type 1 (e.g. Nishapur coloured, Sassanian 1a and 1b) and Type 2 (e.g. Sassanian 2, Nishapur colourless and Samarra) [9].

UU Plant ash Type 1 is characterised by high MgO and K_2_O and low CaO contents. Its high MgO to CaO ratios put it in Mesopotamian Type 2 which matches glasses from Nishapur (colourless glass) and Samarra Groups 1 and 2; they have MgO/CaO ratios ranging from 0.62 to 1.69. This group is distinctive from Mesopotamian Type 1 glasses from Nishapur (coloured glass), which has an intermediate ratio of MgO/CaO and a relatively high content of Al_2_O_3_ (Fig 7).

If we look closely at levels of TiO_2_, Zr, Cr, La and Ce, associated with the silica source, it is possible to refine the provenance and groupings of the ‘Mesopotamian’ glass. In the seminal works on early Islamic plant ash glass, Henderson [5] and Henderson *et al*. [7] have demonstrated that 1000Zr/Ti and Cr/La ratios are particularly effective at separating different groupings of Levantine and Mesopotamian glass from sub-regional production zones. It has become clear that UU Plant ash Type 1 is associated with glasses from Samarra Group 2, characterised by high 1000Zr/Ti ratios and low Cr/La ratios. They are distinct from Samarra Group 1 glasses which have lower ratios of Cr/La, Nishapur glasses, which have lower ratios of 1000Zr/Ti and Cr/La (Fig 9). UU Plant ash Type 1 and Samarra Group 2 can also be distinguished from Samarra Group 1 by their higher concentrations of Zr and Ce. In Fig 8 UU Plant ash Type 1 and Samarra Group 2 have higher Li/K ratios than Samarra Group 1. It is probable that they were produced with different plant species and/or using plants that grew in different geological environments because the elements Li and K are generally introduced in plant ashes [7, 8].

Research by Henderson [5], Henderson *et al*. [7] and Schibille *et al*. [13] have already established scientifically that Samarra was a major primary and secondary glass production centre in the early Islamic period, where glasses were fused from raw materials and shaped into glass vessels and architectural glasses. A possible location where Samarra glasses were made is at al-Qadisiyya, located 25 km from Samarra [13, 43]. Evidence for glassmaking (e.g. furnace glass) at the site was discovered by Brigadier General R. Pope Hennessy in the early 20^th^ century AD. Archival records from the Victoria and Albert Museum (V&A) suggest they came from an ancient kiln. The then V&A curator, B. Rackham, in 1919 remarked that the furnace glass was ‘proof of the site of an ancient (Islamic?) glass works near Samarra’ [43].

Further excavations of the site led to the discovery of a Sassanian-to-Islamic period glass furnace. Evidence for glassworking (e.g. glass waste and glass vessels) was subsequently discovered in the 1986 excavation on site N1 to which historical sources refer as *ma’mal al-zujaj* [5, 13, 43, 57, 58]. Based on the historical, archaeological and scientific evidence, there are reasons to believe that Samarra was a major primary and secondary glass production centre in the early Islamic period. And the similarity between UU Plant ash Group 1 and Samarra Group 2 suggests Samarra, possibly at al-Qadisiyya, was one possible location where UU Plant ash Group 1 was made. Thorough excavations of the site and a proper sampling strategy, especially of raw furnace glass, for scientific analysis would help to define the range of glass compositions made at al-Qadisiyya.

UU Plant ash Types 2 and 3 are characterised by relatively low MgO and K_2_O and high CaO compared to UU Plant ash Group 1 and Samarra Group 2. Fig 7 shows that they have intermediate MgO to CaO ratios and fall into the grouping of Mesopotamian Type 1, consisting of glasses from Siraf Low Zr, al-Qadisiyya, Samarra cobalt blue flasks and three pieces of raw glass from al-Raqqa, which were most likely produced in Iraq/Iran and imported to the city [7]. From Fig 9 it can be seen that there are different regional traditions and production zones for ‘Mesopotamian’ Type 1. UU plant ash Type 2 overlaps with Samarra cobalt blue flasks and low Zr glass from Siraf. UU plant ash Type 2 is distinguishable from the Syrian glass (e.g. six pieces of raw glasses made in al-Raqqa and of Syrian origin) by its lower 1000Zr/Ti ratios and higher Cr/La ratios. It is also separated from other eastern groupings with intermediate Cr/La ratios and lower 1000Zr/Ti ratios. Its concentrations of Zr and Ce are also shown to be higher than the majority of the Siraf Low Zr glass. In Fig 8 UU Plant ash Type 2 is indistinguishable from Siraf Low Zr and the al-Raqqa raw glasses of eastern origin, but it is distinguishable from the Samarra cobalt blue flasks by lower Li/K and Cs/K ratios.

It is not possible to ascertain the provenance of UU Plant ash Type 2. But given its similarity to other Mesopotamian Type 1 glasses, it is certain that UU Plant ash Type 2 originated from multiple glass workshops in the Mesopotamian region including Iraq and Iran. Its similarity to earlier Sassanian 1a and 1b glasses from Veh Ardašīr [59, 60] suggests early ‘Abbasid glassmakers used raw materials with geologically similar or very similar raw materials as Sassanian glassmakers: Ctesiphon, Seleucia and Veh Ardašīr are in more or less the same location [61]. This has already been pointed out for glasses found at Ctesiphon [8] and is also found in UU Plant ash Type 2 and other glass from Siraf and Samarra.

UU Plant ash Type 3 vessels have amongst the highest Al_2_O_3_ and CaO levels and the lowest K_2_O levels. They can also be distinguished from other Mesopotamian Type 1 glasses, including UU Plant ash Type 2, by trace elements associated with both the silica and alkali sources. In Fig 9 it can be seen that UU Plant ash Type 3 is distinguishable from Siraf low Zr glass and Samarra cobalt blue flasks by its high Cr/La ratios and low 1000Zr/Ti ratios. UU Plant ash Type 3 is mainly separated from UU Plant ash Type 2 by its lower 1000Zr/Ti ratios. It is closely related to glasses from al-Qadisiyya, Sassanian 1b and 2 and three fragments of glass from 9^th^– 10^th^ centuries AD Ctesiphon [7, 43, 59, 60]. But its contents of Ce are relatively high (avg. 17 ppm) compared to other Mesopotamian Type 1 glass (avg. Ce concentrations <15 ppm). However, UU Plant ash Type 3 falls mainly with other eastern glass groupings also having low Li/K ratios and high Cs/K ratios and are similar to some other Mesopotamian Type 1 glasses (Fig 8). This suggests the use of similar species of plants from similar geological backgrounds. UU Plant ash Type 3 is unlikely to have come from the Syro-Palestinian region, where glasses are characterised by low Cr/La low Li/K ratios and high 1000Zr/Ti ratios (Figs 8 and 9) [7].

It is not known where UU Plant ash Type 3 originated. Its chemical characteristics suggest a possible eastern provenance. Its similarity to old Sasannian glasses, particularly Sassanian 1b and 2, and al-Qadisiyya again suggests it might have been made in the old Sassanian territories of Iraq and Iran [59, 60]. Curiously, a comparison with four raw glasses from al-Qadisiyya shows some similarities between UU Plant ash Type 3 and the al-Qadisiyya raw glass. Unpublished analyses of furnace glass from a Sassanian–Islamic period furnace at al-Qadisiyya shows that four pieces of the furnace glass identified with Mesopotamian Type 1 glasses based on the MgO/CaO, Li/K, 1000Zr/Ti and Cr/La ratios as well as the concentrations of Al_2_O_3_ in the glasses [43] (Figs 7–9), indicating that al-Qadisiyya was one possible location where UU Plant ash Type 3 and Mesopotamian Type 1 was made in the early Islamic period.

Comparison with contemporary plant ash glass groups shows that GL0213 corresponds to glasses from Nishapur. In most cases, Nishapur glass is markedly different from other Mesopotamian glass with distinctively low 1000Zr/Ti and Cr/La ratios not exceeding 150 and 5.65 respectively (Fig 9). Their contents of Ce are also particularly high, comparable to those from UU Plant ash Types 2 and 3. GL0382 matches glasses from the Levantine group, such as those from Damascus and Khirbat al-Minya as well as Syrian glass from al-Raqqa. They have a rather low Cr/La ratio and high 1000Ti/Zr ratios (Fig 9). GL0382 is well distinguished from the 12^th^– 14^th^ centuries AD glass from Damascus and is closely associated with the 8^th^ century AD Khirbat al-Minya glass. Fig 9 shows that both GL0382 and the Khirbat al-Minya glass have lower 1000Zr/Ti ratios and slightly higher Cr/La ratios than the Damascus glass. However, there are slight differences between GL0382 and the Khirbat al-Minya glass. GL0382 has higher contents of Ce and Li/K ratios than the Khirbat al-Minya glass (Figs 8 and 9), suggesting GL0382 might have been produced with slightly different silica and alkali sources.

From the Unguja Ukuu assemblage, we noted a degree of regional specialisations of glass vessel production in the early Islamic period, assuming, with good reason [7], that in many cases the glass vessels found in cosmopolitan centres like Samarra and al-Raqqa were made on the sites they were found on and that the glass used to make the vessels was fused there too. All of the mid-7^th^– 9^th^ centuries AD ice blue pinched decorated glass were made with Levantine I and UU Plant ash Type 1 (Samarra Group 2) recipe. All (except GL201 (colourless) and GL3461 (turquoise) of the applied trail glass are an olive-green colour. Glass with applied decoration was popular in the eastern Mediterranean area, Syria and Mesopotamia in the early Islamic period. Carboni [62] suggests Syrian glassmakers tended to produce traditional patterns like zigzag and spiralling threads. It is possible that GL2975, decorated with zigzag patterns, was made in Syria with Levantine I glass. On the other hand, other applied trail glass in this study, as well as the blue scratch-engraved glass vessels (GL90, GL2244 and GL2846), were made in Iraq and Iran with Samarra Group 2 and Mesopotamian Type 1 recipes. Both trail and pinch decorations were originated in the Roman and Sassanian periods and continued to be made in the early Islamic period, while scratch-decorated glass is thought to have emerged in the early Islamic period [8, 62].

Certain undecorated glasses fragments with specific characteristics are regionally specific. All of the stepped rims, inward-folded rims as well as blue and turquoise plates belong to UU Plant ash Type 2 and appear to have been made in the eastern region. The triangular-beaked rims, plain fine and plain thick rims which could potentially have formed a range of vessel type were also made in the east with UU Plant ash Types 2 and 3 glass. Three vertical necks (wide) (GL143, GL382 and GL1337) and three folded- ring bases (GL1044, GL1046 and GL1626) were made in Iraq and Iran and belong to UU Plant ash Types 1 and 2. Plain rounded rims, plain thick rims, flaring necks (wide mouth) and rolled-in rims belong to UU Plant ash Type 1 and the vessels that they formed part of were exclusively made in Iraq, possibly in or around Samarra.

Other undecorated glasses are less regionally specific according to their chemical compositions and were made in more than one production zone. The ribbed rim (narrow) vessels were made in the Levantine and the Iraq/Iran region with Levantine I, as well as UU Plant ash Types 1 (Samarra group 2) and 2 glasses. The ice blue plain rounded rims and applied ring bases are specifically made in the Levantine region and possibly in or near Samarra in Iraq. Folded and flattened rims seem to have formed part of another range of popular vessel types, made in both the Levantine and Iraq/Iran region: they were made using all UU plant ash types and Levantine I glass groups. The pushed up bases were made in Egypt and Iraq (e.g. Samarra), though these could have been part of a range of vessel forms

### Trade and globalisation of Islamic glass in the Indian Ocean World in the 7^th^– 9^th^ centuries AD

Our study of early Islamic glass from Unguja Ukuu reveals a complex glass trading and glass supply network between the Middle East and Eastern Africa in the 7^th^– 9^th^ centuries AD. It also serves as a microcosm of the Middle Eastern glass industry in the early Islamic period. The 7^th^– 9^th^ centuries AD saw some of the most radical and fundamental changes in the glass industry in the Middle East, which arguably had a profound impact on the glass trading network and glass supply not just in the Middle East, but beyond in eastern Africa, Southeast Asia and the Far East.

While natron glass continued to be made in Egypt until the late 9^th^ century AD [13], many of the Levantine glass workshops (e.g. Apollonia and Dor) ceased to produce natron glass by the 7^th^ century AD [63]. In the late 8^th^ and 9^th^ centuries AD these were later replaced by a localised glass group Levantine II, which had a much-reduced distribution network [10, 64]. What we see in the Unguja Ukuu glass assemblage and glass assemblages from Middle Eastern sites like al-Raqqa in Syria is that old Levantine I glasses were recycled and reused to produce glass vessels that were characteristically ‘Islamic’ [4, 6, 43]. In the Unguja Ukuu assemblage, Islamic glass vessels and characteristic fragments made with natron glass have been identified. Examples are ribbed rim (narrow) vessels, pinched and trail decorated vessels and folded and flattened rims. The exports of Levantine and Egyptian natron glass also continued, albeit in a much-reduced quantity compared to the previous centuries. Somewhat surprisingly given its date, in Unguja Ukuu, Levantine I glass was more common than Egyptian glass: over 80% of the Unguja Ukuu natron glass were made with Levantine I glass, suggesting either competition between Levantine and Egyptian natron glassmaking centres continued well into the early Islamic period or that trading links were stronger with the Levant due to its being more accessible via the Persian Gulf–even if Levantine I glass has ceased to be made after the 7^th^ century AD.

At the same time, plant ash glass became increasingly dominant in the Middle East and across the world [64, 65]. Demand for and consumption of Islamic plant ash glass increased substantially and expanded outside the Middle East to include East Africa, Southeast Asia and China [8, 66]. In Ramla, the proportion of plant ash glass increased substantially in the 9^th^ century AD, about 85% of the glass was made with plant ash; in al-Raqqa, the proportion of natron glass relative to plant ash glass in the 9^th^ century AD was about 35% to 65%. As a response to the increased demand for plant ash glass in the region, local glass workshops were set up to produce this relatively new type of glass (in the Levant), displacing the natron glass industry. In Ramla, a significant proportion of glass (about 44%) was made in local Levantine workshops such as Tyre, with only a small proportion coming from the eastern workshops such as Nishapur [9]; in al-Raqqa, the majority of the plant ash glass was made locally in al-Raqqa with only a small quantity deriving from the east such as Samarra and Nishapur [6, 7, 43].

Outside of the Middle East, similar findings are observed in the Unguja Ukuu glass assemblage. The proportion of plant ash glass relative to natron glass in the 7^th^– 8^th^ centuries AD Unguja Ukuu was about 65% to 35% in the glass assemblage. By the 8^th^– 9^th^ centuries AD, the proportion of plant ash glass had increased substantially to 85%. It appeared that the supply of glass to east Africa had shifted from the Levantine and Egyptian regions to the Mesopotamian region during the early ‘Abbasid Caliphate, sourcing glass products from workshops such as Samarra, al-Qaddisiya and Nishapur. The presence of Islamic plant ash glass in Unguja Ukuu suggests it was not just a commodity available to regional and local consumers in the Middle East but had rapidly become a widely traded commodity in the broader Indian Ocean and along the maritime and terrestrial Eurasian Silk Road trade network [5, 8]. It also reflects the cosmopolitan and outward-looking nature of Unguja Ukuu (and Swahili) society [67], which established and maintained a strong trade network with the ‘Abbasid Caliphate.

The fact that almost all plant ash glass came from the eastern provinces of the ‘Abbasid Caliphate reflected the importance of the Persian Gulf as a strategic and important commercial gateway to trading communities dispersed in Africa, Southeast Asia and the Far East in the early Islamic period; only one glass object (GL0382) came from the Levantine region. This is not to say that the glass was necessarily produced there, but that it was there that it was organised and collated [24]. Wood et al. [27] have reported that a majority of the plant ash (v-Na-Ca Type A) glass beads from Unguja Ukuu came from the east. They have intermediate 1000Zr/Ti (96–207) ratios and relatively high Cr/La (5.17–11.93) ratios and the majority of which overlap with those from Samarra Group 2, UU Plant ash Type 1 and three of the glass beads overlap with UU Plant ash Type 2. Glass vessel types also indicate a preference for Mesopotamian products. Non-decorated glass vessels such as ribbed neck (narrow) vessels are commonly found in Iraq and Iran, at sites such as Nishapur and Seleucia [24, 68]. Examples of these can also be found in other east African sites such as Manda [69]. Vertical necked (wide) vessels are reportedly found in Nippur and Nishapur and in 8^th^– 10^th^ centuries AD contexts in Shanga and Manda in Eastern Africa [24, 68]. It appears that plant ash glass from the Mesopotamian regions dominated the glass market in Unguja Ukuu, and perhaps Eastern Africa (although more scientific research is needed to test this hypothesis), during the 7^th^– 9^th^ centuries AD–although more scientific research is needed to test this hypothesis.

Looking further east along the Silk Roads, we tentatively suggest the Mesopotamian plant ash glass industry might have had a greater monopoly over the international glass trade than those from the Syro-Palestinian region and Egypt, and such glass was extremely popular among the elites (e.g. in China and Sumatra) who attached high values to eastern glass products [70]. Chemical analysis of Islamic glass from the Famen Temple (9^th^ century AD) in China shows that a great majority of the glass artefacts fall into the Mesopotamian Types 1 and 2 glass group. For instance, a colourless cut vessel and a rim of a colourless vessel were made in the Mesopotamian region, while scratch-decorated glass vessels were possibly made in al-Raqqa and Nishapur [66].

At Lobu Tua in north Sumatra, a majority of the mid-9^th^– 11^th^ centuries AD glass vessels correspond to the so-called Low Zr Group from Siraf, which belongs to the Mesopotamian Type 1 glass group and is said to have been made in Persia. A small number of the vessels appears to overlap with glasses from Nishapur, Sassanian 1a and 1b, and UU Plant ash Type 3 [7, 42, 59, 60, 71]. Similar findings were reported at Angkor, where a majority of the v-Na-Ca (i.e. plant ash) Type 3 glass from Prei Monti correspond to glasses from Lobu Tua [72]. Meanwhile, the late 10^th^ century AD Cirebon shipwreck found off Java was also filled with different types of glass vessels that are characteristically ‘Persian’; examples include pear-shaped ewers with flat handles, bottles with short cylindrical necks, small bottles and vials with square sections or rounded bottoms, faceted, wheel-cut and mould-blown decorated glass, which can be also found at southeast Asian sites such as Lobu Tua and Leran in east Java [73].

The increased domestic and global demand for glass made in Iraq and Iran might explain the diversity of vessel types that have been found across the silk roads compared to those from the Levant and Egypt; these new types appeared to be a market response to the growing demand for Islamic plant ash glass in Eastern Africa, southeast Asia and the Far East. There is no known archaeological and historical evidence to suggest that the glass industry was regulated by the state [5], so this kind of demand was probably met by local producers. Sub-regional eastern glass workshops coincided with cosmopolitan centres, such as Samarra, Ctesiphon and Nishapur. The existence of others is suggested by eastern compositional glass groups but for which we are yet to define their precise geographical locations. These include the Low Zr group and UU Plant ash Types 2 and 3. Basra, an important port at the head of the Persian Gulf, could well have been an important primary glassmaking centre. But we have no secure archaeological or scientific evidence for this. All these compositional groups of glass appear at a time when there was an increased global demand for glass.

The rise of Islamic plant ash glass, particularly those from the Mesopotamian region, coincided with the rapid economic, cultural and political expansion of the ‘Abbasid Caliphate and Islam in the Middle East in the 8^th^– 9^th^ centuries AD. The ‘Abbasids had transferred the centre of Middle Eastern power and economy from the Syro-Palestinian region (previously a major political centre under the Umayyads) to Iraq. An economic boom ensued as a result of these changes and gave rise to the mass production of specific products such as pottery and glass for both export and local consumption [8]. Trade focus had shifted from the Mediterranean to the Mesopotamian region and its trading network expanded into the Indian Ocean and across the Silk Road to China and Japan [43, 74, 75]. This was also connected to the shift in the political centre of the caliphate from Damascus to Baghdad [8].

Islamic culture, religious practices, art and material culture also began to take hold outside the Middle East in this period and foreigners actively engaged with and adopted Islamic culture and practices. Islamic influence on architecture and religious practices in Eastern Africa is readily observed in the form of mosques (e.g. 8^th^– 11^th^ centuries AD Friday mosque at Shanga, possible pre-AD 1000 mosque at Unguja Ukuu), burials (e.g. in simple Islamic style, with bodies laid to rest on their right sides, facing north toward Mecca) and stone houses as early as the 8^th^ century AD [31, 67, 76]. In China, it is well known in historical sources that Muslim traders from the Persian Gulf set up temporary residences in Canton, which was a major trading port for foreign trade in southern China in the 7^th^– 9^th^ centuries AD [77]; there were significant Muslim populations in other Chinese ports, and cities such as Chang’an [5, 78]. In Southeast Asia, at Lobu Tua, Muslim mercantile communities existed as early as the 9^th^ century AD [79].

Islamic pottery, such as 9^th^ century AD Opaque Glazed Wares and mid-9^th^ century AD Splash Glazed Ware, were present at Unguja Ukuu; late 9^th^ or 10^th^ centuries AD lustre decorated, turquoise splashed Opaque Glazed Wares and Early Polychrome Sgraffiato are also well represented in archaeological sites in eastern Africa [26]. The pattern of material remains at Unguja Ukuu and along the Eastern African coast and islands increasingly underscores the influence of Islamic art and material culture in Eastern Africa, and how Unguja Ukuu was at this point integrated into the wider Indian Ocean trade and the Silk Road. And Islamic plant ash glass was part of this global expansion of Islamic art and material culture across the world, integrating workshops and suppliers in the Middle East, trading communities dispersed along the Indian Ocean rim, and consumers in Zanzibar into a complex proto-globalised whole.

## Conclusion

Trace element analysis has allowed us to refine the provenance of glass and the raw materials used to make it. Based on LA-ICP-MS analysis, we have identified two groups of natron glass and three groups of plant ash glass amongst the Unguja Ukuu glass assemblage: UU Natron Types 1, 2 and 3 correspond to Egypt 2 high Na_2_O and Levantine I and II glass groups respectively, which probably originated from Egypt and the Levantine region respectively; UU Plant ash Type 1 corresponds to Samarra Group 2, which are characterised by low Cr/La ratios and high 1000Zr/Ti and Li/K ratios, and which mostly likely originated from Samarra and the glass workshop at al-Qadisiyya, which was located 25 km from Samarra; UU Plant ash Types 2 and 3 have higher Cr/La ratios and Ce and lower 1000Zr/Ti and Li/K than UU Plant ash Type 1. Their chemical characteristics and similarities to old Sassanian glasses suggest a Mesopotamian origin. Our results also reveal a complex glass trading network and glass supply between Unguja Ukku in Eastern Africa and the Middle East. They confirmed that Unguja Ukuu had established strong trade links with the Middle East from the very beginning of its foundation (Crowther et al. in preparation) and was fully integrated into the Islamic glass trade. The Unguja Ukuu glass assemblage also serves as a microcosm of the Middle Eastern glass industry, witnessing the decline of the natron glass industry and the rise and rapid expansion of the plant ash glass industry. Islamic plant ash glass, particularly that made in the Mesopotamian region, was produced in massive quantities to meet the rising demand of Islamic glass across the Middle East, Eastern Africa, southeast Asia and the Far East. It testifies to the appeal of Islamic art and material culture across the Indian Ocean world in the early ‘Abbasid period.

## Supporting information

S1 Table The results of the analysis of SRM612 and BCR-2G standards (expressed in wt% and ppm). (XLSX)

S2 Table LA-ICP-MS results for glass vessels from Unguja Ukuu in Zanzibar. Results are presented in weight percent oxide/element and ppm/element. EPMA results for the major and some minor element oxides (Na_2_O, SiO_2_, CaO, MgO, K_2_O and Al_2_O_3_) and the LA-ICP-MS results for the minor and trace elements.
(XLSX)

S1 Questionnaire (DOCX)
